# Taming the Selection of Optimal Substitution Models in Phylogenomics by Site Subsampling and Upsampling

**DOI:** 10.1093/molbev/msac236

**Published:** 2022-10-28

**Authors:** Sudip Sharma, Sudhir Kumar

**Affiliations:** Institute for Genomics and Evolutionary Medicine, Temple University, Philadelphia, Pennsylvania 19122; Department of Biology, Temple University, Philadelphia, Pennsylvania 19122; Institute for Genomics and Evolutionary Medicine, Temple University, Philadelphia, Pennsylvania 19122; Department of Biology, Temple University, Philadelphia, Pennsylvania 19122; Center for Excellence in Genomic Medicine Research, King Abdulaziz University, Jeddah 21589, Saudi Arabia

**Keywords:** phylogenetics, maximum likelihood, substitution model

## Abstract

The selection of the optimal substitution model of molecular evolution imposes a high computational burden for long sequence alignments in phylogenomics. We discovered that the analysis of multiple tiny subsamples of site patterns from a full sequence alignment recovers the correct optimal substitution model when sites in the subsample are upsampled to match the total number of sites in the full alignment. The computational costs of maximum-likelihood analyses are reduced by orders of magnitude in the subsample–upsample (SU) approach because the upsampled alignment contains only a small fraction of all site patterns. We present an adaptive protocol, ModelTamer, that implements the new SU approach and automatically selects subsamples to estimate optimal models reliably. ModelTamer selects models hundreds to thousands of times faster than the full data analysis while needing megabytes rather than gigabytes of computer memory.

## Introduction

Mathematical substitution models of evolutionary rates between molecular bases and among sites in a multiple sequence alignment (MSA) are among the most fundamental descriptions of molecular evolution (Buckley and Cunningham 2002; Johnson and Omland 2004; Lemmon and Moriarty 2004; Kalyaanamoorthy et al. 2017; Abadi et al. 2020). These models have become invaluable in phylogenetic analyses to track pathogen origins (Boni et al. 2020) and spread (Li, Lai et al. 2021), reconstruct the evolutionary history of genes and species (Kim et al. 2017), and determine the tempo and mode of evolution (Shen et al. 2018). Thousands of research articles report selecting the optimal substitution model (Darriba et al. 2012; Kalyaanamoorthy et al. 2017; Tamura et al. 2021) using Bayesian and other information criteria (Hurvich and Tsai 1989; Posada and Crandall 1998; Kalyaanamoorthy et al. 2017) to compare the Maximum-Likelihood (ML) fit of several nested and non-nested substitution models.

**Fig. 1.**
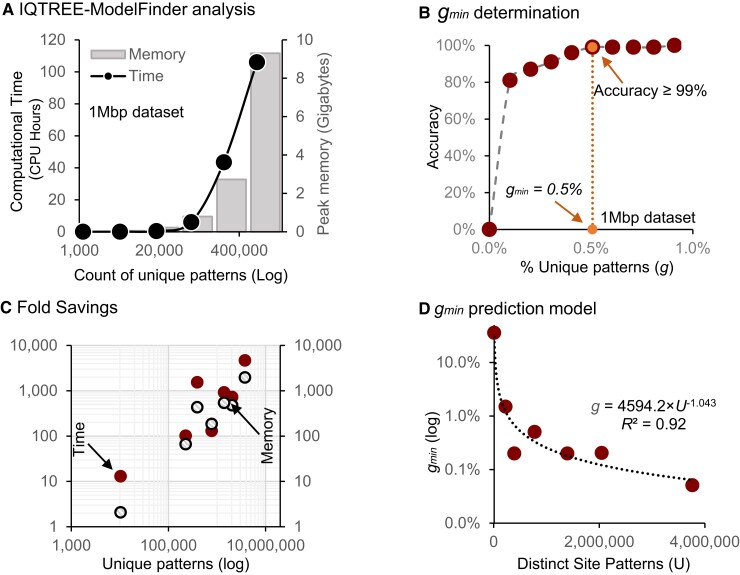
Accuracy and computational resource required for model selection using the SU datasets. (*A*) Increase in computational time (dots) and memory (bars) for analyzing sequence alignment with increasing numbers of distinct site patterns (log scale) sampled from the 1 Mbp dataset. (*B*) The accuracy of model selection for subsampled–upsampled (SU) datasets for different fractions of unique site patterns (*g*) sampled from the 1 Mbp dataset. The accuracy is the percentage of SU datasets for which the model selected was the same as that for the full MSA. The dotted line marks the point (*g*_min_ = 0.5%) at which the accuracy becomes 99%. (*C*) Fold savings in computational time and memory were achieved in SU analysis of many large datasets for subsamples of size *g*_min_ at which the accuracy was at least 99% (table 1). (*D*) The power relationship between the number of total unique patterns (*U*) and the fraction of site patterns needed (*g*_min_) for ≥ 99% accuracy in model selection.

The computational needs of model selection analyses grow exponentially with the acquisition and assembly of increasingly longer sequence alignments (Kapli et al. 2020; Sharma and Kumar 2021). For example, IQ-TREE's ModelFinder (IQ-MF) needed 9.3 GB of computer memory (RAM) and more than four days of computing (CPU time) to evaluate 286 models needed to select the optimal substitution model for concatenated DNA sequence alignment from 37 mammals (*L* = 1,391,742 sites; hereafter 1 Mbp dataset) (Song et al. 2012). This is because the computational costs are a function of the total count of unique site patterns (*U*) in the whole alignment (fig. 1*A*) (Sharma and Kumar 2021). Partitioning the 1 Mbp dataset by codon positions also produced very long alignments (each >460,000 sites) that required more than 3.6 GB of RAM and 55 CPU hours of computing for model selection. In our survey of recently published articles using phylogenomics, we found that scientists routinely compare results from the analysis of both concatenated and partitioned datasets (Prasanna et al. 2020; Vasilikopoulos et al. 2020; Haelewaters et al. 2021; Li, Steenwyk et al. 2021). In these analyses, model selection for concatenated sequences and long partitions can require many hours of computing and up to gigabytes of computer memory (table 1).

**Table 1.** Time and Memory Requirements of Optimal Model Selection.

Data Summary					Full MSA			Subsample–upsampled (SU) dataset				
Data	Type	Sequences	All Sites	Unique Patterns	Memory (GB)	Time (Hours)	Optimal Model	Patterns Used	*g*_min_	Accuracy	Memory (GB)	Time (Hours)
**Butterflies**	DNA	61	5,267,461	3,762,723	75.0	3,140.7	GTR + F + R	1,918	0.05%	100%	0.04	0.67
**Insects A**	DNA	174	3,011,544	2,045,783	115.4	5,000.3	GTR + F + R	4,183	0.20%	99%	0.24	6.85
**Insects B**	DNA	48	2,938,039	1,396,402	21.7	656.0	GTR + F + R	2,796	0.20%	99%	0.04	0.71
**Mammals**	DNA	39	1,391,742	775,579	9.3	106.0	GTR + F + R	3,930	0.51%	99%	0.05	0.81
**Yeasts**	AA	23	634,530	390,960	13.0	973.5	LG + F + R	783	0.20%	100%	0.03	0.63
**Birds**	DNA	200	394,684	226,490	14.6	258.0	GTR + F + R	3,389	1.50%	100%	0.22	2.53
**Simulated**	DNA	52	12,300	10,348	0.1	0.1	TIM2 + F + R	3,709	36.0%	99%	0.06	0.01

**Note.−** *g*_min_ is the minimum fraction of unique site patterns required for selecting the optimal model with an accuracy ≥ 99% in the subsample–upsampled analysis. All model selection analyses were conducted using ModelFinder in IQ-TREE. *g_min_* values were rounded up to two digits after the decimal point.

## Results

### The Approach of Upsampling Sites From Subsamples

In the 1 Mbp dataset, the number of distinct site patterns (*U* = 775,579) is orders of magnitude larger than the number of free parameters in the most complex substitution model evaluated by IQ-MF. From this observation, we hypothesized that a faction of site patterns (*g*) is likely sufficient to infer the optimal model reliably, that is *g* < 100%. If true, this property will enable computational efficiency of the order 1/*g* in both time and memory for ML analyses. To test this hypothesis, we empirically determined the smallest *g* that consistently produced the optimal substitution model identical to that selected using the full MSA by using IQ-MF. We constructed 100 phylogenomic subsamples of the 1 Mbp dataset, each containing 1% of the unique site patterns (*g* = 1%). Subsamples were constructed by selecting sites randomly without replacement from the 1 Mbp alignment until the subsample contained *g × U* different site patterns. Before applying IQ-MF to this phylogenomic subsample, we expanded it by randomly upsampling its sites until the new alignment contained as many sites as the original MSA. Specifically, sites were selected randomly with replacement from the subsample until the total number of sites became the same as the full MSA (Kleiner et al. 2014; Sharma and Kumar 2021). Therefore, the subsample–upsample (SU) dataset contained 1,391,742 sites, equal to the number of sites in the 1 Mbp dataset. We surmised that an SU dataset would have statistical power similar to the full MSA's in selecting the optimal model for large enough *g*. The proportion of SU datasets that produced the same optimal model as the full MSA is the accuracy of the SU approach for the given *g*. This accuracy was 100% for SU datasets with *g* = 1% when using IQ-MF for both SU and full MSA analyses.

The 1% SU dataset contains a small fraction of unique site patterns but has the same number of total sites as the full MSA. This means that every site pattern occurs many times in the SU dataset. Because the time and memory needs of the ML analysis are a function of the number of unique site patterns rather than the total sequence length, the analysis of the 1% SU dataset was 100 times faster and required proportionately less memory. On average, SU datasets utilized only 94 megabytes of peak RAM and 1.4 CPU hours.

### Minimum Subsample Size for Efficient Model Selection

Experimenting with phylogenomic subsamples of the 1 Mbp dataset, we found that a high model selection accuracy could be achieved for even smaller subsamples (fig. 1*B*). Accuracies ≥99% were observed for *g* ≥ 0.5%, that is the minimum fraction of unique site patterns (*g*_min_) needed to select an optimal model reliably for the 1 Mbp dataset was 0.5%. This analysis required only 42 megabytes of peak RAM and was 130 times faster (0.81 vs. 106 CPU hours). In contrast, the analysis of subsamples *without* upsampling had a low accuracy (12%) for *g* = 0.5%. The performance of phylogenomic subsampling *without* upsampling may not be improved through post hoc linear transformations of the information criteria (e.g., BIC) to account for the underrepresentation of the number of substitutions in the subsample because such linear adjustments may not change the relative ranks of the models tested.

**Fig. 2.**
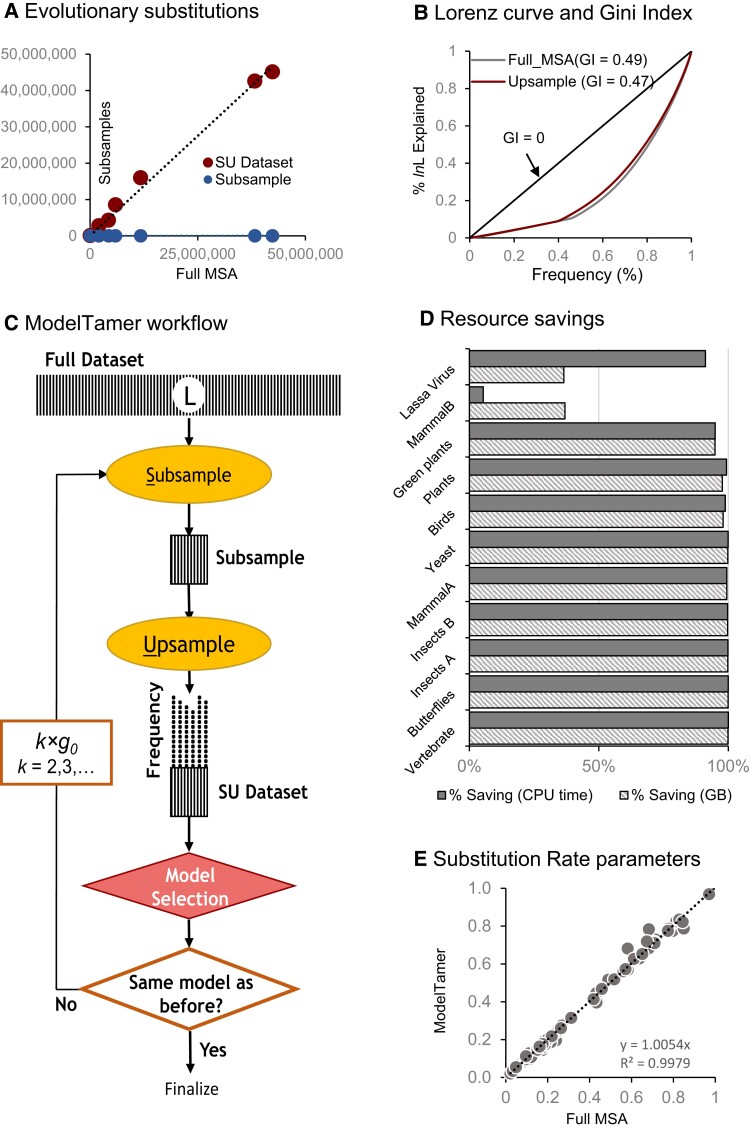
ModelTamer protocol and performance in model selection. (*A*) Relationships between the total number of substitutions in the full MSAs and their SU datasets (dotted line; slope = 1.1) and subsample-only datasets (dots on the x-axis; slope = 3 × 10^−6^). (*B*) The Lorenz Curve for the relationship between the frequencies of site patterns and the proportion of the overall log-likelihood (lnL) contributed by those site patterns for 1 Mbp full MSA (lower curve) and the SU dataset with *g* = 0.5% (higher curve). The Gini Index (GI) shown measures the inequality of information content distributed among site patterns. (*C*) Flowchart of ModelTamer analysis. The shaded box represents the original sequence alignment containing sequences of length ***L***, which has ***U*** unique site patterns. A subsample (small, shaded box) contains a specified fraction (*g*) of unique site patterns from the full MSA. The initial value of *g* is predicted using the trend in panel 1*D*. A random sample of sites is drawn with replacement (multinomial sampling) from the subsample, which is then upsampled. The upsampled dataset has the same number of sites (*L*) as the full MSA, but the number of unique site patterns remains the same as the subsample. Each site pattern is represented many times in the SU dataset, represented by many black dots above each position in the shaded box (SU dataset). The model selection is performed on this SU dataset. The optimal model found in this analysis can be validated by building SU datasets containing an increasing number of unique site patterns (*k* × *g*, *k* = 2, 3, …) until two consecutive runs produce the same optimal model. (*d*) Computational savings in memory (GB) and time (CPU hours) achieved by ModelTamer for large and small empirical datasets (see table 2). (*e*) Scatter plot showing the relationship of the estimated instantaneous substitution rate between bases from full MSA and SU analysis (slope ∼1.0) for empirical DNA datasets in table 2.

Therefore, the upsampling procedure can overcome the analytical limitations of phylogenomic subsamples by achieving higher accuracy without increasing the computational burden. This is because the numbers of unique site patterns are almost the same in datasets with and without upsampling, but the total number of evolutionary substitutions in the SU datasets was similar to that in the full MSAs for the 1 Mbp dataset (fig. 2*A*; ratio = 0.99). Also, the Lorenz curve and the Gini index for the SU dataset were similar to the full MSA (fig. 2*B*), showing that SU datasets recapture the pattern of information contents among the site patterns in the full MSA. This result suggests that the upsampling procedure ensures the inclusion of sufficient counts of different types of base substitutions to select the optimal model reliably. This was not the case for site subsamples without upsampling (ratio = 0.000003; fig. 2*A*), which results in a lower accuracy than SU datasets (12% vs. 95%).

We estimated *g*_min_ for many large empirical datasets from diverse species (butterflies, insects, birds, and yeasts) to assess and measure the generality of the pattern observed for the 1 Mbp dataset (table 1). These MSAs contained 23–200 sequences and as many as 3.7 million distinct site patterns. For large datasets, accuracy ≥ 99% was achieved with *g*_min_ < 1%, saving >98% of computational time and memory (table 1). For many of these datasets, > 1,000× computational efficiency was achieved (fig. 1*C*). Generally, *g*_min_ was smaller for longer sequence alignments (fig. 1*D*; *R*^2^ = 0.92).

### Adaptive Tool for Model Selection

We implemented the SU method into an adaptive tool (ModelTamer) that automatically determines the minimum *g* and selects the optimal model for use in empirical data analysis (fig. 2*C*). ModelTamer can be used with any method for selecting the optimal model, for example IQ-MF, jModelTest (Darriba et al. 2012), ModelTest-NG (Darriba et al. 2020), and MEGA-CC (Kumar et al. 2012). ModelTamer first calculates the initial fraction of site patterns (*g_0_*) to subsample, which is predicted using the relationship between *g*_min_ and *U* shown in figure 1*D*. Then, it subsamples *U* × *g_0_* unique site patternss by random sampling of sites without replacement from the full MSA. The SU dataset is then generated by upsampling in which the subsample is augmented by randomly sampling sites from the starting subsample. The SU dataset is analyzed in the next step using the chosen model selection method (IQ-MF here). The optimal model for the SU dataset can be based on BIC, AIC, AICc, likelihood ratio test, or another statistical criterion. In the next step, the number of patterns subsampled is increased to 2*×g_0_*. Optimal models produced by the analysis of *g_0_*-SU and *2×g_0_*-SU are then compared. If they do not match, then the subsample size is expanded (*k* × *g*_0_, *k* = 3, 4, …) and model selection is applied. ModelTamer stops when two consecutive analyses produce the same substitution model (fig. 2*C*). We implemented ModelTamer coupled with IQ-MF in an R program, which also gives users the flexibility to further validate the selected model by increasing the number of site patterns in the SU dataset.

We applied ModelTamer with IQ-MF to many large and small empirical datasets and found it to produce the same model as the IQ-MF analysis of the full MSAs (table 2). ModelTamer realized ≥95% saving in computational memory and time for large empirical datasets, as the estimated $\;{\hat{g}}_{\min}$ from 0.1% to 2.4% (table 2, fig. 2*D*). These savings are expected to be smaller for datasets that contain a small number of unique site patterns because *ModelTamer* will need to use a larger fraction of site patterns in each subsample to include a few thousand unique site patterns necessary for a reliable substitution model selection (table 2, fig. 2*D*). In all these analyses, ModelTamer did not select the same optimal model as the full MSA for one small empirical DNA dataset (Lassa Virus; table 2). For this dataset, ModelTamer selected a model that was the second best in the IQ-MF analysis of the full MSA. Interestingly, the difference in BIC between the top two models was less than 10, which means that these two model fits will be considered statistically indistinguishable (Kalyaanamoorthy et al. 2017). This suggests that for smaller datasets, ModelTamer may sometimes produce a different but statistically equivalent model to that produced by the analysis of the full MSA.

**Table 2.** Performance of Model Selection by *ModelTamer* for Empirical and Simulated Datasets.

Data	Bases	Sequences	All Site Patterns	Used Patterns	${\hat{g}}_{min}$	Optimal Model (MT)	Memory (GB)	Time (Hours)	Memory Saving	Time Saving
***Empirical Datasets***										
** Big Datasets**										
Butterflies	DNA	61	3,762,723	3,810	0.1%	GTR + F + R	0.08	1.00	99.9%	99.97%
Insects A	DNA	174	2,045,783	4,190	0.2%	GTR + F + R	0.24	7.20	99.8%	99.9%
Vertebrates	AA	58	1,547,914	1,806	0.1%	JTT + F + R	0.16	3.50	99.9%	99.9%
Insects B	DNA	48	1,396,402	4,217	0.3%	GTR + F + R	0.07	1.95	99.7%	99.7%
Mammals A	DNA	39	775,579	4,702	0.6%	GTR + F + R	0.06	0.63	99.4%	99.4%
Yeasts	AA	23	390,960	783	0.2%	LG + F + R	0.03	1.00	99.8%	99.9%
Birds	DNA	200	226,490	4,504	2.0%	GTR + F + R	0.29	3.10	98.0%	98.8%
Plants	DNA	16	190,352	4,615	2.4%	GTR + F + R	0.02	0.13	97.7%	99.3%
** Small datasets**										
Green plants	AA	360	17,789	883	5.0%	JTT + F + R	0.51	10.10	94.9%	94.9%
Mammals B	DNA	274	4,303	2,710	63.0%	GTR + F + R	0.24	1.95	36.9%	5.3%
Lassa Virus	DNA	179	1,475	931	63.0%	GTR + F + R*	0.05	0.03	36.5%	91.2%
										
***Simulated Datasets***										
** Big Datasets**										
This article #1	DNA	50	95,852	26,895	28.1%	JC	0.44	0.41	71.9%	47.2%
This article #2	DNA	50	95,820	31,508	32.9%	K2P	0.51	0.75	67.2%	−0.04%
This article #3	DNA	50	92,600	21,916	23.7%	HKY	0.36	1.15	76.3%	33.2%
This article #4	AA	20	43,864	5,794	12.6%	Poisson	0.18	0.38	86.8%	92.2%
This article #5	AA	20	44,895	5,840	13.3%	WAG	0.18	0.39	87.0%	86.5%
This article #6	AA	20	46,327	8,624	18.5%	JTT	0.26	1.22	81.6%	78.1%
** Small Datasets**										
Abadi et al. 1	DNA	44	13,110	2,612	19.9%	TPM2u + F + R	0.08	0.02	59.4%	86.4%
Abadi et al. 2	DNA	51	10,348	4,336	41.9%	TIM2 + F + R	0.05	0.06	70.8%	27.3%
Kalyaanamoorthy et al. 1	AA	100	9,806	994	10.0%	LG + R	0.16	1.96	86.5%	93.6%
Kalyaanamoorthy et al. 2	AA	100	9,781	995	10.0%	LG + R	0.16	1.98	89.7%	96.0%
Kalyaanamoorthy et. al. 3	AA	100	9,775	993	10.0%	LG + R	0.16	2.00	89.7%	93.4%
Abadi et al. 3	DNA	52	7,442	4,524	60.8%	HKY + F + R	0.08	0.02	39.2%	88.0%

**Note.** All empirical datasets analyzed were gathered from published articles. We simulated big datasets (see *Material and Methods*) and used existing ones from published articles. ${\hat{g}}_{\min}$ is the estimated fraction of unique site patterns needed by *ModelTamer* for selecting the optimal model. Peak memory and total time used in the ModelTamer analysis are shown. Time and memory savings are the percent reductions compared to the full MSA analysis. The selected model by *ModelTamer* was the same as the full MSA analysis, except for one dataset (*) for which alternative statistically indistinguishable models existed (BIC difference < 10) (Kalyaanamoorthy et al. 2017). All savings estimates used the time and memory taken for IQ-MF analysis of full MSA. ${\hat{g}}_{\min}$ values were rounded up to one digit after the decimal point.

The optimal models selected by IQ-MF for large empirical datasets were usually the most complex models tested, so ModelTamer also selected complex models. We examined ModelTamer's performance when the actual underlying substitution process was simple, but the sequences were long. We generated such DNA sequence datasets by computer simulations in which the simplest Jukes and Cantor (1969) (JC) nucleotide substitution model was used. IQ-MF on the full MSA and ModelTamer with IQ-MF selected the JC model (table 2). ModelTamer analysis of sequence alignments produced by computer simulations under models with additional parameters, such as Kimura (1981) 2-parameter (K2P) and Hasegawa-Kishino-Yano (1985) (HKY) models, also produced correct models (table 2). ModelTamer also worked well in finding the optimal model produced by the IQ-MF analysis of the full MSA for three simulated DNA datasets obtained from Abadi et al. (2020) (table 2). In these analyses, ModelTamer frequently offered high memory and time savings (table 2).

We also produced amino acid (AA) sequence alignments by simulating datasets in which the instantaneous substitution rates between AA residues were the same (Poisson model). ModelFinder produced the correct model (table 2). We also tested ModelTamer's ability to distinguish among equally complex AA substitution models by analyzing sequence alignments simulated using the JTT model (Jones et al. 1992), WAG model (Whelan and Goldman 2001), and LG model (Le and Gascuel 2008) (see ***Material and Methods***). Both IQ-MF with full MSA and ModelTamer worked well (table 2). ModelTamer saved more than 75% of computational time and memory in these analyses. Based on these analyses, we expect the accuracy of ModelTamer in selecting the correct optimal model to be the same as that of the tool used in *ModelTamer* for evaluating the fit of different models (e.g., IQ-MF) because the ModelTamer system is intended to reduce the time and memory needs of model selection faithfully through site subsampling and upsampling. ModelTamer can be coupled with any method, including methods that consider data errors introduced during molecular sequencing and sequence alignments (Spielman and Miraglia 2021).

### ModelTamer for Partitioned Datasets

In the above, we have presented the efficiency of ModelTamer for sequence alignments in which all the genes and genomic segments were concatenated for phylogenomic analysis. In addition to concatenated MSA, most systematic studies also designate collections of sites (partitions) based on biological, functional, and/or genomic considerations. ModelTamer can be applied to each partition separately to select the best-fit model efficiently. The adaptive nature of ModelTamer will automatically use all the site patterns for shorter partitions, taking no more time and memory than the standard tool used for model selection. For shorter sequence lengths, ModelTamer achieved 5–95% savings for computing time and 36–95% of peak RAM (table 2, fig. 2*D*). ModelTamer will offer high memory and time savings for longer partitions, such as those based on genome source (e.g., mitochondrion, chloroplast, and nucleus) (Kimball et al. 2021), specific codon positions (Dos Reis et al. 2018), functional annotations (e.g., coding and noncoding) (Thode et al. 2020; Kimball et al. 2021), and prior biological and evolutionary features (Prasanna et al. 2020; Vasilikopoulos et al. 2020; Haelewaters et al. 2021). Of course, one may eliminate the expense of model selection by simply using the most complex substitution model, but this approach has been debated in the literature (Keane et al. 2006; Hoff et al. 2016; Abadi et al. 2020). For AA sequence analysis, however, many models are equally complex and would require model selection for which ModelTamer is efficient and accurate. Furthermore, as shown below, ModelTamer greatly reduces the time and memory needed for estimating substitution rate matrix parameters for any model for long sequences.

### Estimating Model Parameters by SU Analysis

For a long AA sequence alignment from 58 vertebrate species (1,806,035 sites), IQ-MF required 4,604 CPU hours (6.4 CPU months) to finish the optimal model selection on a high-performance computer with 139 GB of RAM. For this dataset, ModelTamer analysis required less than 3 h and less than 1 GB of memory (*g*_min_ = 0.1%), making optimal selection feasible. The time required was orders of magnitude less than IQ-MF's for even fitting a given substitution model and a fixed phylogeny to this dataset, as IQ-MF needed 69 GB of RAM and 130 CPU hours. Interestingly, estimates of the substitution rates and other model parameters (e.g., mean relative rate) produced by ModelTamer were very similar to those from the analysis of full MSA. ModelTamer estimates of the substitution rate matrix parameters showed a 1:1 relationship with those produced from the analysis of empirical DNA datasets (fig. 2*E*, slope > 0.99; *R*^2^ > 0.99). ModelTamer's estimates of site-wise substitution rates were also close to those from full MSA analysis (slope = 0.96–1.00; *R*^2^ ≥ 0.99).

## Conclusions

The power of upsampling of site subsamples and its desirable theoretical properties are already known for estimating confidence intervals (Kleiner et al. 2014; Sharma and Kumar 2021). Here, we have demonstrated that only a small representative fraction of unique site patterns contains sufficient information to effectively select the optimal substitution model and estimate its rate parameters. We have also shown that a simple protocol (ModelTamer) can automatically determine the fraction of site patterns necessary for SU analysis. These findings are likely to have implications for the general application of the SU approach. Ultimately, we expect ModelTamer to reduce the enormous computational demands of model selection that precede big data phylogeny inference for which many efficient tools exist (Stamatakis 2014; Nguyen et al. 2015; Sharma and Kumar 2021). Consequently, researchers with even commodity computers will be able to conduct big data analysis on their desktops, and those utilizing high-performance computing infrastructure will benefit by achieving greater calculation parallelization because of the small memory footprint of individual calculations in ModelTamer. These computational efficiencies will promote higher scientific rigor, broader participation, and environment-friendly computing in molecular evolutionary research (Kumar 2022).

## Materials and Methods

### Empirical and Simulated Sequence Data Assembly

Eleven empirical DNA and AA sequence alignments were analyzed from yeasts (Salichos and Rokas 2013), plants (Ran et al. 2018), insects (A (Peters et al. 2017), and B (Peters et al. 2018)), butterflies (Allio et al. 2020), birds (Prum et al. 2015), mammals (A (Song et al. 2012), and B (dos Reis et al. 2012)), Lassa viruses (Andersen et al. 2015), green plants (Ruhfel et al. 2014), and jawed vertebrates (Chen et al. 2015) (tables 1 and 2). Five empirical datasets (butterflies, birds, Insects A and B, mammal A, and yeast) and one simulated dataset were used to generate the *g*_min_ prediction model. The number of species ranged from 16 to 360, and the number of sites ranged from 3,186 to 5,267,461.

We also analyzed DNA and AA sequence alignments gathered from published research articles (Kalyaanamoorthy et al. 2017; Abadi et al. 2020) (tables 1 and 2) and new simulations to generate datasets with specific properties. DNA sequence alignments were simulated using simple models: Jukes-Cantor (1969) model (JC), Kimura (1981) 2-parameter model (K2P), and Hasegawa-Kishino-Yano (1985) model (HKY). The transition versus transversion rate ratio for both K2P and HKY models was set to 2.00, and the base frequencies for the HKY model were set to be (A = 31%, C = 27%, G = 20%, and T = 22%) referring to HKY + F model in IQTREE (Nguyen et al. 2015). Each simulated DNA sequence alignment contained 50 sequences with a sequence length of 100,000 (table 2). Similarly, a set of AA datasets were simulated under an equal substitution probability (Poisson model) and more complex models: JTT (Jones et al. 1992) and WAG (Whelan and Goldman 2001). The AA sequence alignments simulated were 50,000 long and contained 20 sequences. For simulating each sequence alignment, a random tree was generated using an R function (-rtree) from the *ape* package, where the branch length varied uniformly between 0 and 0.2. The multiple sequence alignments were simulated using IQTREE (*–alisim* option). The ground truth for these simulated datasets was the substitution modes determined by analyzing full sequence alignment using IQ-MF (Nguyen et al. 2015; Kalyaanamoorthy et al. 2017).

### Model Selection Analysis

We used ModelFinder in IQ-TREE (IQ-MF) with default options to select the optimal model in all analyses, skipping the advanced search option (-mtree) due to excessive computational time requirement as this option uses a separate initial tree for each of the models tested. One hundred subsample–upsampled (SU) datasets were generated for each *g* (0.1–1%). The accuracy is the proportion of times SU datasets selected the same optimal model as the full MSA using IQ-MF. The *g*_min_ is the minimum *g* needed to achieve accuracy ≥99%. Accuracy was also calculated for subsamples in which no upsampling was performed. We chose IQ-MF because it is now widely used in empirical data analysis. Other approaches, such as jModelTest (Posada 2008; Darriba et al. 2012; Nguyen et al. 2015), were tested and produced similar relative computational savings. Unfortunately, our attempts to use machine learning methods (Abadi et al. 2020) for large datasets were not always fruitful because of the absence of machine learning methods for AA sequence alignments and the failure of all available online/offline tools to produce optimal models for large nucleotide sequence alignments.

### ModelTamer Analysis

We used the ModelTamer protocol (fig. 2*C*) implemented in R (R Core Team 2020). This package has a customized function, “SU_MSA,” to generate SU datasets using the “Biostrings” package (Pagès et al. 2017). IQ-MF (Kalyaanamoorthy et al. 2017) was applied to each SU dataset; one can couple other tools for model selection with ModelTamer. We expect the relative resource-saving to be similar when using other tools because the cost of ML analysis is a function of the unique site patterns used in all the software packages. The “aggregator_model” function processes all the outputs and provides the optimal model and its parameters. It also outputs peak memory usage and the CPU time required by *ModelTamer*. We have also developed an automated function (*ModelTamer*.R) in R described in the main text, which takes the sequence alignment as input for model selection and produces the optimal substitution model and its parameters.

## Data Availability

All empirical and simulated datasets analyzed in this article are gathered from published articles. These empirical sequence alignments are from yeast, viruses, plants, insects (A and B), butterflies, birds, eutherian mammals, and jawed vertebrates. These empirical and simulated datasets are available in the figshare repository (Sharma and Kumar 2022). R functions and the automated pipeline for model selection are available from https://github.com/ssharma2712/ModelTamer. A description file with an example dataset for implementing each function of *ModelTamer* is provided in the repository.
